# Novel amino-ethyl carboxymethyl cellulose crosslinked ampholyte hydrogel development for Methyl orange removal from waste water

**DOI:** 10.1038/s41598-024-64245-8

**Published:** 2024-06-26

**Authors:** Ahmed M. Omer, Wagih A. Sadik, Rafik Abbas, Tamer M. Tamer, Mai M. Abd-Ellatif, Mohamed S. Mohy-Eldin

**Affiliations:** 1 Polymer Materials Research Department, Advanced Technology and New Materials Research Institute (ATNMRI), City of Scientific Research and Technological Applications (SRTA-City), New Borg El-Arab City, Alexandria, 21934 Egypt; 2 Department of Materials Science, Institute of Graduate Studies and Research, Alexandrian University, Alexandria, Egypt

**Keywords:** Methyl orange, Adsorption, Aminated carboxymethyl cellulose, Ampholyte hydrogel, Dye removal, Water treatment, Environmental sciences, Chemistry, Materials science

## Abstract

In the modern era, with the rapid growth of various industries, the issues of energy crisis and environmental pollution have garnered increasing attention. One significant source of industrial pollution is printing and dyeing wastewater. This wastewater often contains dyes that have aromatic structures and azo groups, such as Methyl orange (MO), which are both toxic and difficult to degrade. If these dyes are released into the wastewater stream without any treatment, they can have adverse effects on ecological balance and human health. Therefore, it is crucial to identify suitable treatment strategies to efficiently remove dyes from wastewater systems before discharge. In this study, the Methyl orange (MO) azo dye has been removed from dyes-contaminated wastewater, for the first time, using a novel amino-ethyl carboxymethyl cellulose crosslinked ampholyte hydrogel (AECMC). Different characterization methods, including FTIR, TGA, and DSC were used to characterize the generated AECMC compounds. The water absorption and cationic exchange capacities were assessed. Factors affecting the MO anions adsorption including MO concentration, adsorption pH, temperature, time, adsorbent dose, and agitation speed have been investigated. Moreover, the kinetics of the adsorption process was assessed by the use of three models: pseudo-first-order, Pseudo-second-order, and Elovich. Moreover, the mechanism of the adsorption process was monitored using the Intraparticle diffusion and Boyd models. Additionally, the adsorption isotherm was examined using established models such as Langmuir, Freundlich, and Temkin isotherms. The thermodynamic characteristics of the MO adsorption process have been investigated at various adsorption temperatures using the Van't Hoff model. The results obtained from the study indicate that the process of MO adsorption adhered to the Pseudo-second-order kinetic model, the Langmuir isotherm model was found to be applicable, and spontaneous and exhibited an endothermic character. In conclusion, the developed novel amino-ethyl carboxymethyl cellulose crosslinked ampholyte hydrogels (AECMC) have successive in the removal of the MO anionic dye from contaminated wastewater.

## Introduction

Methyl Orange (MO) is a water-solution azo dye used in research labs and the textile sector^1^. Regardless of the capacity of some gut bacteria to metabolize the aromatic amine groups^2^, the dye is present at a high concentration (5 g/L), and it is not commonly degraded by hydrolysis in polluted waters^3^. Accordingly, removing it from aqueous solutions is challenging using current water treatment techniques. As a result, MO lingers in the environment if improperly managed and poses a risk to living things^4^. Due to dyes' carcinogenic and mutagenic properties, dye leakage into wastewater has been highlighted as a factor influencing human health^5^. Physical, chemical, and biological processes have successfully removed synthetic dyes. Some techniques include membrane separation, biological degradation, enhanced oxidation, and adsorption^6^. Because the structures of synthetic dyes are refractory and complicated, conventional procedures are frequently used to break down these pigments in wastewater^7^. Recent research has shown that the adsorption approach is advantageous because of its widespread availability, reduced cost, and ability to be recycled^8^. Commercial adsorbents, including charcoal, minerals, and polymer complexes, have limited active adsorption sites, reducing their effectiveness^8^. As a result, numerous studies have been conducted on utilizing and enhancing the effectiveness of biosorbents' dye removal. These biosorbents are more eco-friendly and efficient at adsorbing dyes. According to reports, biosorption is efficient for maximizing color removal from dye-contaminated fluids^9^.

In this direction, Polysaccharide-based hydrogels emerged as promising adsorbents especially for removing synthetic dyes from industrial waste^10,11^. Due to the presence of different functional groups such as –OH, –COOH, and –NH2 in polysaccharide structure makes them suitable candidates for the adsorption of different dyes^11,12^. Hydrogels have applications in diversified fields such as drug delivery, water purification, desiccant materials, and soft-tissue engineering^13–15^. Most hydrogels can adsorb either cationic or anionic dyes. Anionic polysaccharides (e.g. xanthan gum, karaya gum, etc.) based hydrogels can be used to adsorb only cationic dyes efficiently, whereas, cationic polysaccharides (e.g. Chitosan (CS)) based hydrogels is a cationic polysaccharide which has –NH2 functional groups in abundance can be used in the treatment of anionic dyes contaminated wastewater^16–19^. However, carboxymethyl cellulose (CMC) is an anionic polysaccharide having carboxymethyl groups in its structure^20^, and its hydrogels have shown potential to treat cationic dyes contaminated wastewater^21^. Therefore, targeting the adsorption of both cationic as well as anionic dyes using a single adsorbent is a quite challenge. This limitation can be overcome by using different approaches such as; (a) a combination of cationic and anionic polysaccharides in the hydrogel synthesis-based matrices or composite adsorbents^22–27^, (b) modification of the chemical structure by grafting and formation of composites^28–30^, (c) formation of composite using inorganic materials^31^, and (d) Formation of ampholytes^32–34^.

In this study, we adopted a simple approach to develop carboxymethyl cellulose ampholyte hydrogel through amination of the hydroxyl groups with 2-chloroethyl amine using click chemistry followed by chemical crosslinking using Glutaraldehyde. The adsorption process of the anionic MO dye has been investigated, for the first time, under various operational conditions, including temperature, pH, initial MO concentration, adsorption period, adsorbent dosage, and agitation speed. Utilizing various methods like FTIR, TGA, and DSC, the developed new amino-ethyl carboxymethyl cellulose crosslinked ampholyte hydrogel (AECMC) derivatives have been characterized. The water absorption percentage and the cationic exchange capacity have also been assessed and connected to the adsorption efficiency. Moreover, the adsorption process has been studied using different kinetic, isotherm, and thermodynamic models.

## Materials and methods

### Materials

Carboxymethyl Cellulose (CMC, With Molecular Weight (90,000), DS = 0.7), was obtained From Across Organics Company—New Jersey (USA) in the form of Sodium Salt. 2-Chloro ethyl amine and Whatman ® qualitative filter paper, Grade 1, were obtained from Sigma-Aldrich (Germany). Sulfuric acid 98%, Sodium hydroxide, and phenol phethalin were purchased from El-Nasr Pharmaceutical Co. for Chemicals (Egypt). Glutaraldehyde (GA, 25.0 wt% solution in water) obtained from ACROS Organics.

### Methods

#### Preparation of amino-ethyl carboxymethyl cellulose crosslinked hydrogel (AECMC)

In 40 mL of distilled water, 2 g of CMC were dissolved. Next, several concentrations of Cl-ENH_2_ (6.4, 12.7, 25.5, and 51 mM) were added. The temperature was then elevated to 70 °C for three hours while stirring. 0.5 mL of 25% Glutaraldehyde was added while stirring continuously for an hour. The obtained AECMC hydrogel was overnight dried at 60 °C, then grounded using a ball mill, sieved (200 µm), and repeatedly rinsed in distilled water to remove unreacted components. According to the concentration of Cl-ENH_2_, the powder was finally dried and coded as AECMC-1, AECMC-2, AECMC-3, and AECMC-4, then stored in desiccators until use.

#### Adsorption batch equilibrium studies

Methyl orange (MO) dye was made as a stock aqueous solution at a concentration of 1g/L (1000 ppm), from which the desired concentrations were obtained by diluting with distilled water. All of the adsorption studies were carried out in 100 mL flasks by introducing a predetermined amount of adsorbent to 25 mL dye solutions with varying dye concentrations and a predetermined pH value, then shaking for the predetermined amount of time in an orbital shaker (100 rpm). The hydrogel adsorbents were filtrated using filter paper. Each experiment was repeated at least three times and the standard error was found ± 5%. The MO concentrations in the initial and final aqueous solutions were determined using a UV–Vis spectrophotometer set to 465 nm through a standard curve of MO concentration ranging from 2 to 20 ppm of pH 7.0. For the variation of the adsorption pH study, the absorbance of MO at each pH solution before adsorption has been established as a reference using 20 ppm MO concentration. The difference between the initial and equilibrium concentrations was used to compute the amount of dye adsorbed. The following relationships were used to calculate the values of adsorption capabilities:

1 $${\text{Adsorption capacity }}\left( {{\text{mg}}/{\text{g}}} \right) = \left[ {\left( {{\text{C}}_{0} - {\text{C}}_{{\text{t}}} } \right) \, \times {\text{ V}}} \right]/{\text{M}}$$

where V is the volume of the dye solution (L), C_t_ is the final dye concentration in the supernatant (mg/L), and M is the mass of the adsorbent (g).

#### Characterization

##### Infrared spectrophotometric

Infrared spectra were performed with Fourier transform infrared spectrophotometer (Shimadzu FTIR—8400 S, Japan) to confirm modification and prove the structure of hydrogel. Samples were mixed with KBr to make pellets. FT-IR spectra in the transmittance mode were recorded using an FT-IR spectrometer (Shimadzu FTIR- 8400 S, Japan), connected to a PC, and analysis the data by IR Solution software, Version 1.21. The spectra (128 scans at 2 cm^−1^ resolution) were collected with a frequency range of 4000–400 cm^−1^. The FTIR spectra were Fourier-deconvoluted with a resolution enhancement factor of 1.5 and a bandwidth of 15 cm^−1^.

##### Thermal gravimetric analysis (TGA)

Analysis by TGA of samples was carried out using a thermo gravimetric analyzer (Shimadzu TGA-50, Japan) under Nitrogen flow (30 mL/min) to evidence changes in structure as a result of the modification. The weight loss of the samples was measured starting from room temperature to 600 °C at a heating rate of 10 °C/min.

##### Differential scanning calorimeter (DSC)

Differential scanning calorimetric analysis of the samples was carried out using a Differential Scanning Calorimeter device (Shimadzu DSC–60A, Japan) in temperatures ranging from ambient to 350 °C at a heating rate of 10 °C/min under nitrogen flow (30 mL/min).

##### Morphological characterization (SEM) and energy dispersive X-ray analysis (EDAX)

With the help of an analytical scanning electron microscope (Joel Jsm 6360LA, Japan), SEM micrographs of the AECMC adsorbents were obtained. In addition to morphological inspection, the composition (mass ratios) of the AECMC and AECMC-MO adsorbents were evaluated by EDAX analysis in conjunction with scanning electron microscopy to assess the concentration of added-on amine and sulfonic groups.

##### Water uptake

The produced hydrogel's swelling behavior was examined using distilled water. Hydrogels were precisely weighed out and submerged in water before being left to swell for 24 h at 37 °C. The swelled hydrogel was periodically separated, and the moisture that clung to their surface was removed by gently blotting them between two filter papers, which was done just after weighting. Each experiment was repeated at least three times and the standard error was found ± 5%. The following formula was used to calculate the samples' swelling levels^35^.

2 $${\text{Water uptake}\;}\left( \% \right) = \left[ {\left( {{\text{M}}_{{\text{t}}} - {\text{M}}_{0} } \right)/{\text{M}}_{0} } \right] \times {1}00$$

where, M_t_ is the weight of the swollen hydrogel, and M_0_ is the initial dry weight.

##### Ion exchange capacity of hydrogel

A known weight of the amino-ethyl carboxymethyl cellulose crosslinked hydrogels (AECMC) was added to the known volume of 0.1 M H_2_SO_4_ solution, and the mixture was kept under shaking for three hours. The mixture was filtered using Whatman filter paper (grade 1), and an aliquot was titrated against a standard solution of sodium hydroxide. Similarly, control titration without the addition of carboxymethyl cellulose was also run. From the difference in the volume of NaOH required for neutralization, the ionic capacity of the AECMC sample was calculated using the following equation:

3 $${\text{Ion exchange capacity}} = \left( {{\text{V}}_{{2}} - {\text{V}}_{{1}} } \right){\text{a}}/{\text{w }}\left( {{\text{meq}}/{\text{g}}} \right)$$

where V_2_ and V_1_ are the volumes of NaOH required for complete neutralization of H_2_SO_4_ in the absence and presence of the AECMC, A is the normality of NaOH, and W is the weight of the AECMC sample taken for analysis^36^. Each experiment was repeated at least three times and the standard error was found ± 5%.

##### Reusability

The AECMC-4 adsorbent (0.1 g) was used in the adsorption process of MO (20 ppm) from a 25 mL dye solution of pH 7.0 at 25 °C for 60 min. After completion of the adsorption process, the MO-AECMC-4 adsorbent was separated using Whatman filter paper (grade 1) and subsequently washed repeatedly using distilled water, then transferred to a 100 mL beaker containing 25 mL of 0.1N NaOH solution. The solution was stirred at 25 °C for 6 h using a 400 rpm stirring rate. The regenerated AECMC-4 adsorbent was separated as mentioned above and then washed repeatedly with distilled water to remove all remaining NaOH to reach pH 7.0 of the washing water, then dried at 60 °C overnight. The dried regenerated AECMC-4 adsorbent was used in another adsorption cycle under the same adsorption conditions. This adsorption–desorption cycle was repeated ten times.

## Results and discussions

### Adsorbent characterization

#### Infrared spectrophotometric (FT-IR)

**Figure 1:**
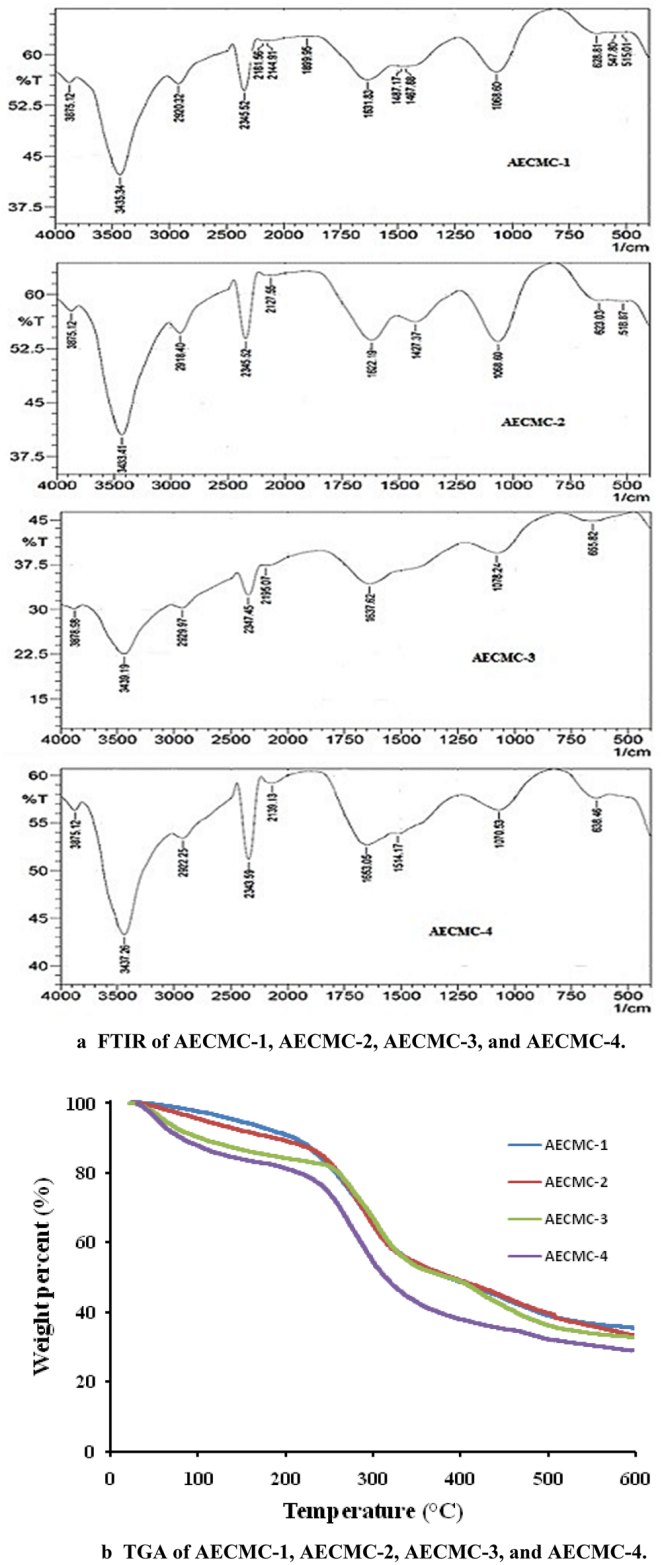

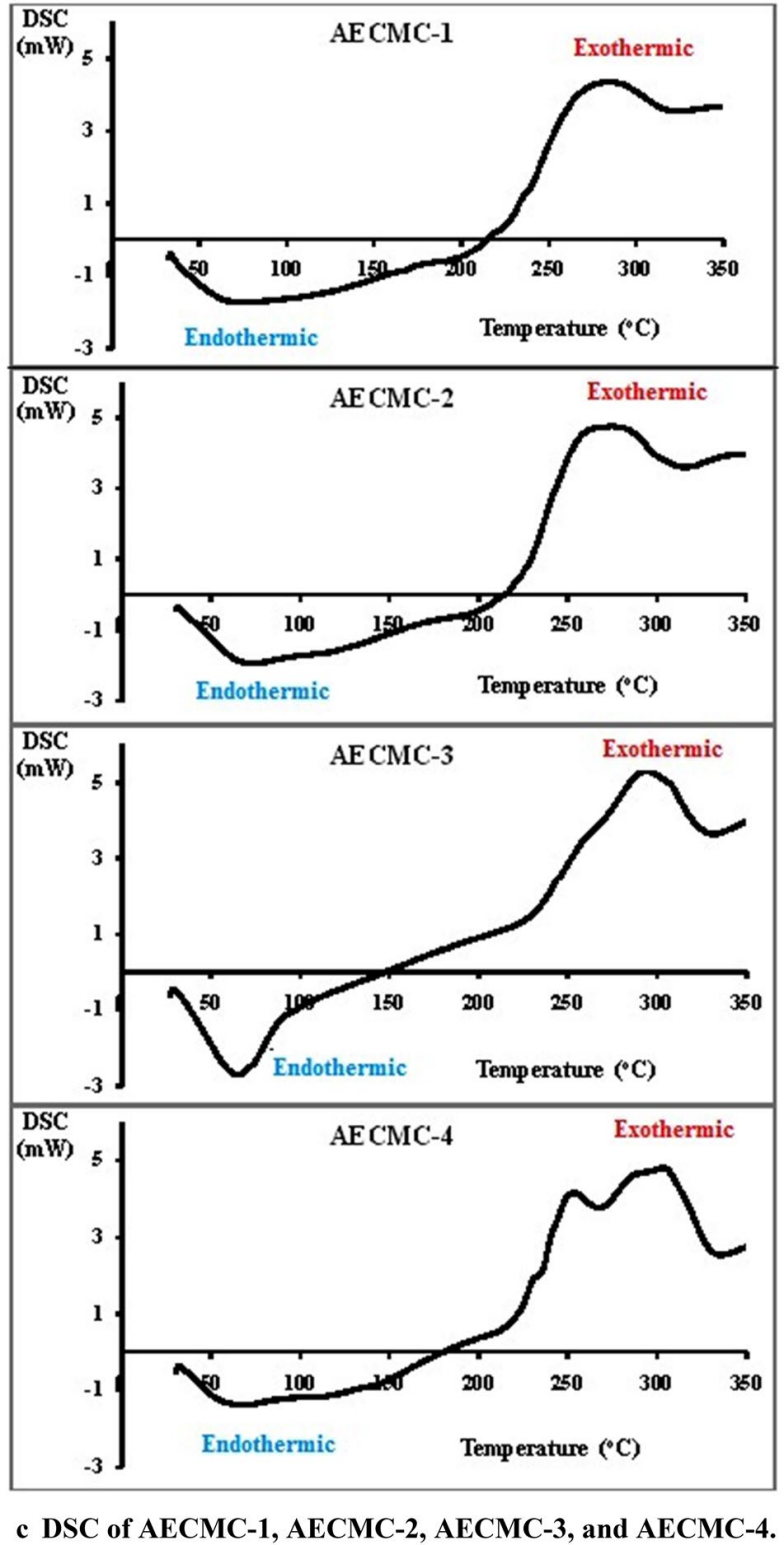
(**a**) FTIR of AECMC-1, AECMC-2, AECMC-3, and AECMC-4. (**b**) TGA of AECMC-1, AECMC-2, AECMC-3, and AECMC-4. (**c**) DSC of AECMC-1, AECMC-2, AECMC-3, and AECMC-4.

FTIR spectroscopy was broadly utilized in cellulose investigation as it affords a simplistic way of getting direct information on chemical changes that happen through different chemical treatments^37^. Figure 1a demonstrates FT-IR spectra of AECMC hydrogels with different content of ethylene amine. The spectra display the characteristic peaks of cellulosic function groups^38^, such as O–H stretching (hydrogen Bond) at 3438 cm^−1^, and the band at 1487 cm^−1^ in the spectra indicates the OH bending of the adsorbed water^39^. The bands around 1467 cm^−1^ and 1325 cm^−1^ are assigned to CH2 scissoring and OH bending vibration, respectively. Moreover, the IR spectra display the presence of the carboxyl, C=O group at 1622–1637 cm^−1^. According to^40^, carboxyl groups and their salts show two peaks at the wave number about 1600–1640 cm^−1^ and 1400–1450 cm^−1^ that symbolize the presence of carboxymethyl substituent. The band at 2920 cm^−1^ was due to C-H stretching and at 1427 cm^−1^ absorbance, reflecting the bending of C–C–C. Band 1068 cm^−1^ refers to C–O stretching vibration. The grafting of ethyl amine along the CMC chain does not give a characteristic change in FT-IR spectra that can be explained by the presence of amine groups in the same regain of CMC function groups.

#### Thermal gravimetric analysis (TGA)

Thermal gravimetric analysis (TGA) is a technique employed to describe the thermal stability of polymeric materials through the relation between weight change and temperature (Fig. 1b). The initial degradation step of AECMC adsorbents around 100 °C was attributed to the loss of moisture content due to evaporation^41^. The charts record increases in weight loss as 5.2% for AECMC-1, 7.78% for AECMC-2, 13.32% for AECMC-3, and 16.09% for AECMC-4 confirm the increase in the hydrophilic nature of hydrogel by increase immobilized amine groups according with the obtained results of the ion exchange capacity in Fig. 1. The water uptake (%) shows a diverse behavior, as shown later, which referred to the increase of the crosslinking degree of the formed AECMC hydrogels confirming the increase of the adherent water content to the introduced amine groups in favor of the entrapped water in the three-dimensional hydrogel structures. The second weight loss, starting from 240 to 400 °C, is due to the loss of carbon dioxide from carboxymethyl groups of CMC and the destructive decomposition of the glucopyranose ring^42^.

#### Differential scanning calorimetry (DSC)

DSC analysis of the AECMC hydrogels was measured and presented in Fig. 1c. The first endothermic peak for all tested samples, which was begun from ambient to around 120 °C, was attributed to the release of the trapped moisture molecules. Hydrophilic groups along CMC, such as hydroxyl and carboxylic groups along the polymer backbone, give it a high affinity for water that can be trapped in the surrounding atmosphere or during preparation conditions. In addition, the amination of the CMC increases its hydrophilic nature. The second thermal event of the chart was of the AECMC hydrogels, which may be related to the decomposition of the glucopyranose ring units with correspondence exothermic peak at 250 °C and degradation decomposition of the carboxylic group^43^. As a result of the amination process, the exothermic peak has been shifted to a higher temperature close to 300 °C with increased sharpens of the AECMC-1, AECMC-2, and AECMC-3 adsorbents. A split of the exothermic peak of the AECMC-4 adsorbent has been observed and distinguish strong peak at 250 °C was recognized. Two other merged peaks have been observed in the temperature range 280–310 °C which may be referred to the formation of poly(ethyl amine) grafted branches due to the excess of used Cl-ENH_2_ concentration (51 mM), which is double the concentration used in preparation of the AECMC-3 adsorbent, rather than monolayer attachment of ethyl amine molecule to the CMC backbone OH groups.

#### Scanning electron microscope (SEM) and EDAX analysis

**Figure 2:**
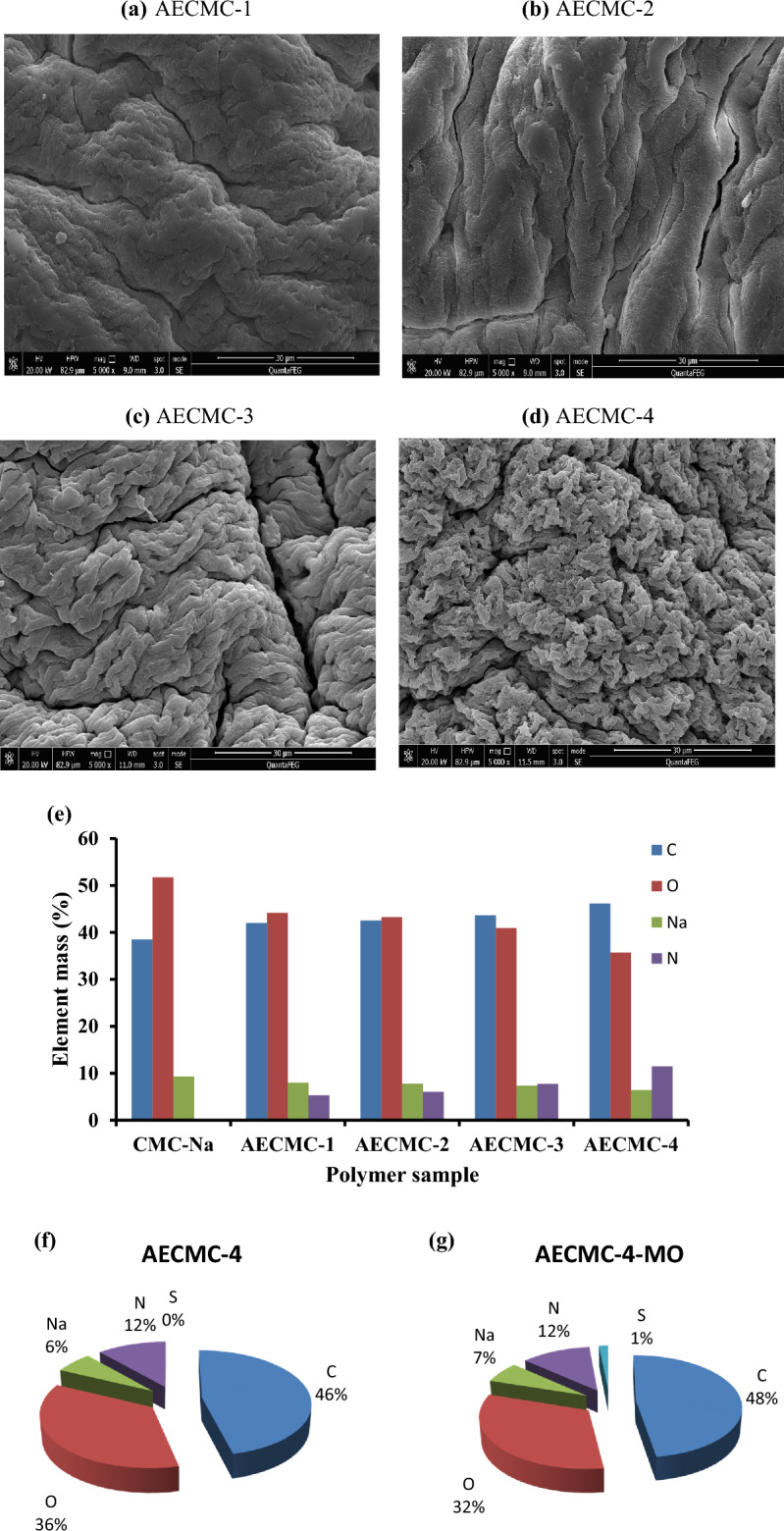
SEM pictures of AECMC adsorbents and EDAX analysis of AECMC-4 and AECMC-4-MO adsorbents.

Figure 2 displays the microstructure of the surfaces of AECMC adsorbents. It observed the influence of increasing the immobilized amine groups on the morphological structure of adsorbent. AECMC-1 (Fig. 2a) adsorbent has a merged filaments structure combined with smooth flacks on the surface and noticeable cracks. With further increase of the immobilized amounts of amine groups (Fig. 2b–d), the filament structure starts to be more precise, and the size of the filament is reduced with more rough cracked surfaces. AECMC-4 adsorbent (Fig. 2d) of the highest amine content shows the smallest filaments with high observed homogenous porosity. That can be referred to as the presence of long side chains with a terminal amine. These new side chains increase the spacing between polymer chains. In addition, it provides an active site for crosslinking with Glutaraldehyde.

The immobilization of amine groups has been verified through EDAX analysis (Fig. 2e), where the C mass percent has been increased along with the N mass percent in benefits of Na and O mass percent of the CMC-Na blank sample. Figure 2f,g provide evidence of MO adsorption on the AECMC-4-MO (Fig. 2g) compared to AECMC-4 adsorbent (Fig. 2g) through the show up of the S element and increased the C and Na mass percent in benefits of O mass percent. In contrast, the N mass percent remained unchanged.

### Effect of adsorption time

The effect of adsorption time on the adsorption capacity of the developed AECMC adsorbents using different concentrations of 2-chloro-ethylamine to remove MO from contaminated wastewater has been investigated. The results are tabulated in Table 1. From the Table 1, two main observations have been noticed. The first is the increase of the adsorption capacity with the adsorption time for all the developed adsorbent matrices. The second is the higher the obtained adsorption capacity as much as the higher the 2-chloro-ethylamine concentration used in the amination of CMC. It is clear that the adsorption capacity of the AECMC-1 adsorbent has been almost unchanged value after 150 min. For other adsorbents; AECMC-2, AECMC-3, and AECMC-4, the adsorption capacity is constant starting from 180 min for the first two adsorbents (AECMC-2 and AECMC-3), while a little change observed for the last one; AECMC-4. The adsorption process lasted for 210 min to reach equilibrium.

**Table 1:** Effect of the adsorption time on the adsorption capacity of the developed AECMC adsorbents.

Adsorption capacity (mg/g)	Adsorption time (min)							
	15	30	60	90	120	150	180	210
AECMC-1	0.51 ± 0.026	0.585 ± 0.029	0.658 ± 0.033	0.813 ± 0.041	0.94 ± 0.047	**1.01** ± 0.051	**1.01** ± 0.051	**1.04** ± 0.052
AECMC-2	0.755 ± 0.038	0.928 ± 0.046	1 ± 0.05	1.258 ± 0.063	1.29 ± 0.065	1.345 ± 0.067	*1.4* ± 0.07	*1.41* ± 0.071
AECMC-3	0.92 ± 0.046	1.03 ± 0.052	1.188 ± 0.059	1.43 ± 0.072	1.52 ± 0.076	1.52 ± 0.076	***1.62*** ± 0.081	***1.62*** ± 0.081
AECMC-4	1.12 ± 0.056	1.323 ± 0.066	1.425 ± 0.071	1.52 ± 0.076	1.67 ± 0.084	1.725 ± 0.086	1.735 ± 0.087	1.76 ± 0.088

Significant values are given in bold, italic, bold italic and underlined.

For better understanding, the ion exchange capacity of the developed AECMC-1, AECMC-2, AECMC-3, and AECMC-4 adsorbents, 3.5, 4.0, 5.1, and 7.5 meq/g, has been correlated with the water uptake (%), 321.8, 268, 215, and 103.5, and the adsorption capacity obtained at equilibrium, 210 min; 1.04, 1.41, 1.62, and 1.76 mg/g, respectively. The data shows that the increase of the IEC to 5.1 meq/g has a positive linear effect on the adsorption capacity. After that, the adsorption capacity increment reduced than expected (2.42 mg/g) by about 27% (1.76 mg/g). This behavior could be attributed to the negative impact of reducing the water uptake of the developed AECMC adsorbents resulting from crosslinking the introduced amine groups via reaction with Glutaraldehyde^18^. This effect impacts the diffusion of MO molecules to reach the protonated amine groups only in the case of the AECMC-4 adsorbent. That behavior could be attributed to other factors related to reducing the concentration gradient between the soluble MO molecules in the liquid phase, and its concentration adsorbed onto the AECMC-4 adsorbent solid phase^16–18^.

### Effect of dye concentration

Table 2 shows the effect of variation of the MO concentration on the adsorption capacity of the developed AECMC adsorbents. Table 2 shows two observations that depend on the immediate relation between the dye concentration and the available binding sites on an adsorbent surface^44^. The first observation is that the increase of the initial dye concentration up to 50 mg/L (ppm) causes a linear increase in the adsorbent's loading capacity, which may be due to the high driving force for mass at a high initial dye concentration^45^. That indicates a high number of active sites relative to the number of MO molecules in the liquid phase of all the MO concentrations used where free active sites are still available. The second observation is the adsorption capacity leveling off with the increase of the initial MO concentration above 50 mg/L (ppm), where equilibrium was almost reached. That indicates the reduction of the available active sites relative to the number of MO molecules in the liquid phase of the MO concentrations above 50 mg/L (ppm). It is worth mentioning here that at a specific MO concentration, the adsorption capacity increases with the adsorbents’ ion exchange sites (IEC) increase. However, this increment is not a linear one. For example, the adsorption capacities of AECMC-3 and AECMC-4 are very close regardless of the ion exchange capacities values: 5.1 meq/g and 7.5 meq/g, respectively. That behavior could be explained according to the created surface ion exchange sites (amine groups), which are higher in the case of AECMC-4 than its counterpart, AECMC-3. Accordingly, the MO concentration gradient between the liquid and polymer phases was reduced, and the equilibrium was reached.

**Table 2:** Effect of the dye concentration on the adsorption capacity of the developed AECMC adsorbents.

Adsorption capacity (mg/g)	Dye concentration (ppm)					
	10	20	30	50	70	100
AECMC-1	0.476 ± 0.024	0.526 ± 0.0263	0.798 ± 0.040	1.07 ± 0.054	1.1 ± 0.055	1.04 ± 0.052
AECMC-2	0.704 ± 0.035	0.804 ± 0.040	1.1 ± 0.055	1.36 ± 0.068	1.47 ± 0.074	1.42 ± 0.071
AECMC-3	0.896 ± 0.045	0.95 ± 0.048	1.31 ± 0.066	1.67 ± 0.084	1.722 ± 0.086	1.74 ± 0.087
AECMC-4	1 ± 0.05	1.14 ± 0.057	1.46 ± 0.073	1.71 ± 0.086	1.82 ± 0.091	1.86 ± 0.093

### Effect of adsorption temperature

Table 3 shows the effect of variation in the MO adsorption temperature on the adsorption capacity. From the Table, it is clear that elevation of the adsorption temperature has a linear, direct, positive effect on the MO adsorption capacity. This effect is inversely related to the IEC of the AECMC adsorbents. The increment percentages of the adsorption capacity with increasing adsorption temperature from 25 to 60 °C were found as follows: 124%, 38%, 29%, and 17.5% for AECMC-1, AECMC-2, AECMC-3, and AECMC-4 adsorbents, respectively. The enhancement behavior upon temperature elevation indicates the endothermic nature of the MO adsorption process. This behavior may be referred to the effect of temperature on water uptake (%) of the adsorbents, which facilitates both the dye molecules' adsorption processes on the surface and the diffusion to interior pores of the adsorbent hydrogel. The progressive swelling with temperature increases the accessibility of the interior amine groups to the diffusive MO molecules, which keeps the concentration gradient between the dye aqueous phase and the adsorbent solid phase enough as the driving force of the adsorption process.

**Table 3:** Effect of the adsorption temperature on the adsorption capacity of the developed AECMC adsorbents.

Adsorption capacity (mg/g)	Adsorption temperature (°C)				
	25	30	40	50	60
AECMC-1	0.658 ± 0.033	0.788 ± 0.039	0.86 ± 0.043	1.2 ± 0.060	1.48 ± 0.074
AECMC-2	1 ± 0.050	1.14 ± 0.057	1.27 ± 0.064	1.318 ± 0.066	1.38 ± 0.069
AECMC-3	1.19 ± 0.060	1.23 ± 0.062	1.44 ± 0.072	1.493 ± 0.075	1.54 ± 0.077
AECMC-4	1.43 ± 0.072	1.46 ± 0.073	1.56 ± 0.078	1.64 ± 0.082	1.68 ± 0.084

### Effect of adsorption pH

**Figure 3:**
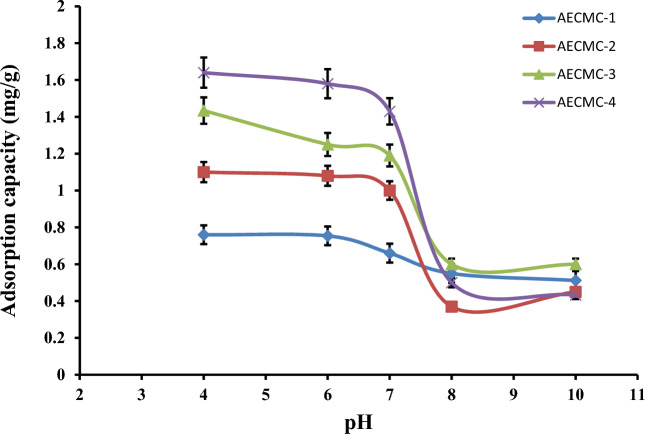
Effect of the adsorption pH on the adsorption capacity of the developed AECMC adsorbents; 25 mL of 20 ppm MO, 180 min, 30 °C, 100 rpm, and 0.1 g adsorbent.

The effect of the pH value of initial MO solutions on adsorption is shown in Fig. 3. The dye uptakes are much more significant in acidic solutions than in alkaline conditions. At acidic pH, free amine groups along the AECMC backbone are protonated, forming a positive charge on the hydrogel surface that promotes electrostatic interaction with the negative charge of MO (i.e., negatively charged sulfonate group). In this region, adsorption is controlled by electrostatic physical- and chemisorption. The similar results are also mentioned in published results^46,47^. At alkaline pH, the adsorption capacities of AECMC hydrogels are declined. In this pH, the surface charge of AECMC adsorbents is negative, which hinders the adsorption by the electrostatic force of repulsion between the negatively charged dye molecule and AECMC adsorbents. However, an appreciable amount of dye removal in this pH range suggests a strong involvement of physical forces, such as hydrogen bonding, van der Waals force, etc., in the adsorption process^48^.

### Effect of adsorbent dose

**Figure 4:**
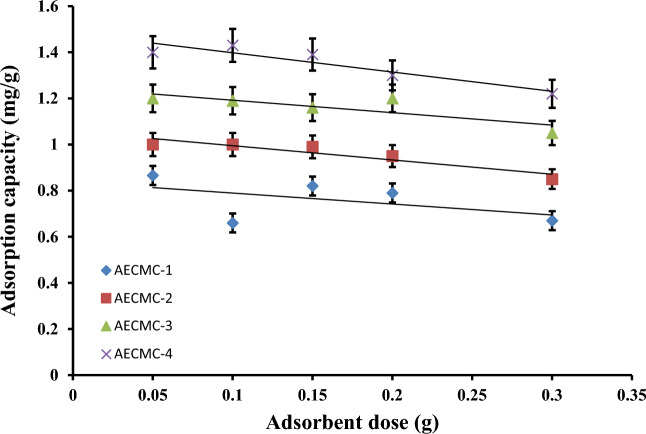
Effect of the adsorbents dose on the adsorption capacity of the developed AECMC adsorbents; 25 mL of 20 ppm MO, pH 7.0, 180 min, R.T, and 100 rpm.

The effect of variation in the adsorbent dose on the adsorption capacity has been investigated, and the obtained results are presented in Fig. 4. The Figure shows that the adsorption capacity for the developed AECMC adsorbents decreased linearly with increasing the adsorbent dose. The reduction percentage of the adsorption capacities ranged from 12.5% of AECMC-3 to 23% of AECMC-1 adsorbents. That behavior may be explained by the fixed number of MO dye molecules in the solution, while the number of active sites increases with the adsorbent dose. However, it is worth mentioning that the MO removal percentages were found to increase with the adsorbent dose for all the AECMC adsorbents, as seen in Table 4. The highest MO removal percentage reached 73% of AECMC-4 adsorbent. It is interesting to mention that at any specific adsorbent dose studied, both the adsorption capacity and MO removal percentages increased linearly with the amine content from AECMC-1 to AECMC-4 adsorbents.

**Table 4:** Effect of the adsorbents dose, (g), on the MO removal percentages of the developed AECMC adsorbents.

Adsorbent code	Adsorbent dose (g)				
	0.05	0.1	0.15	0.2	0.3
AECMC-1	8.66	13.137	24.89	31.62	39.96
AECMC-2	10.14	20.076	30.41	37.94	50.96
AECMC-3	12.35	23.757	35.19	48.15	63.15
AECMC-4	14.14	28.512	42.1	51.78	73.41

### Effect of agitation speed

The agitation speed variation from 100 to 250 rpm has almost no significant effect on the adsorption capacity of all the developed AECMC adsorbents. The only exception was found in the case of the AECMC-1 adsorbent, where the adsorption capacity increased by 25% with increased agitation speed within the studied range. That behavior could be explained by the lower percentage of the active adsorption sites over the adsorbent surface to the interior adsorption active sites.

### Reusability

**Figure 5:**
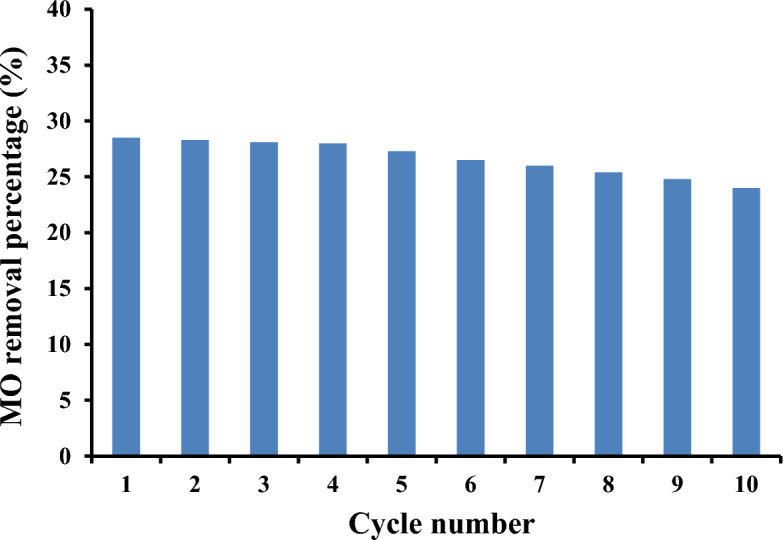
Reusability of the AECMC-4 adsorbent in removal of MO dye.

An estimate of the extent of recovery of MO absorbed from aqueous solution can be made employing the sorption desorption cycle, as illustrated in Fig. 5. In this respect, the sorption–desorption cycles were analyzed to investigate the AECMC-4 adsorbent reusability for removing MO from aqueous solutions. The cycle was performed ten times by using a sodium hydroxide solution. The figure clearly shows that a continuous, almost linear reduction has been observed after the fourth cycle. However, the reduction in the removal efficiency of MO was not drastic; the MO removal percentage reached 24% in the tenth cycle compared with 28.5% in the first one. Only 16% of the MO removal efficiency was lost after ten cycles of sorption–desorption processes. The AECMC-4 adsorbent has a good sorption–desorption performance and can be used confidently without a noticeable reduction in its sorption capacity to remove MO.

### Comparative adsorption capacity study

A comparison of the maximum adsorption capacity for MO on AECMC-4 adsorbent with other adsorbents reported in the literature^49–53^ is shown in Table 5.

**Table 5:** Comparison of the adsorption capacity of AECMC-4 adsorbent with other adsorbents.

Adsorbent	Adsorption capacity (mg/g)	References
Chitosan	19.92	^19^
Chitosan/4-dimethylamino benzaldehyde Schiff base	19.34	
Chitosan/benzophenone Schiff base	22.37	
Calcium alginate MWNTs	13.1	^50^
Magnetic MWCNTs	13.1	^51^
Chitosan	8.867	^18^
Chitosan/succinimide Schiff base	10.0	
Chitosan/1-methyl-2-pyrrolidinone Schiff base	7.2	
Chitosan	5.54	^17^
Chitosan/4-methoxybenzaldehyde Schiff base	7.73	
Chitosan/organic rectorite	5.0	^49^
Bottom ash	3.618	^53^
Alginate/polyaspartate hydrogels	0.22–0.28	^52^
Alginate	0.08–0.28	^52^
Aminated ethyl carboxymethyl cellulose (AECMC-4)	1.8	This study

According to the data tabulated, the adsorption capacity is higher than alginate and alginate/poly aspartate hydrogels^52^. The case was reversed when Calcium alginate MWNTs were used^50^, where the adsorption capacity was six times higher. On the other hand, the adsorption capacity of AECMC-4 is relatively low compared with other Chitosan and Chitosan derivatives^17–19,49^. Magnetic MWCNTs and Bottom ash show higher adsorption capacity, too^51,53^.

**Figure 6:**
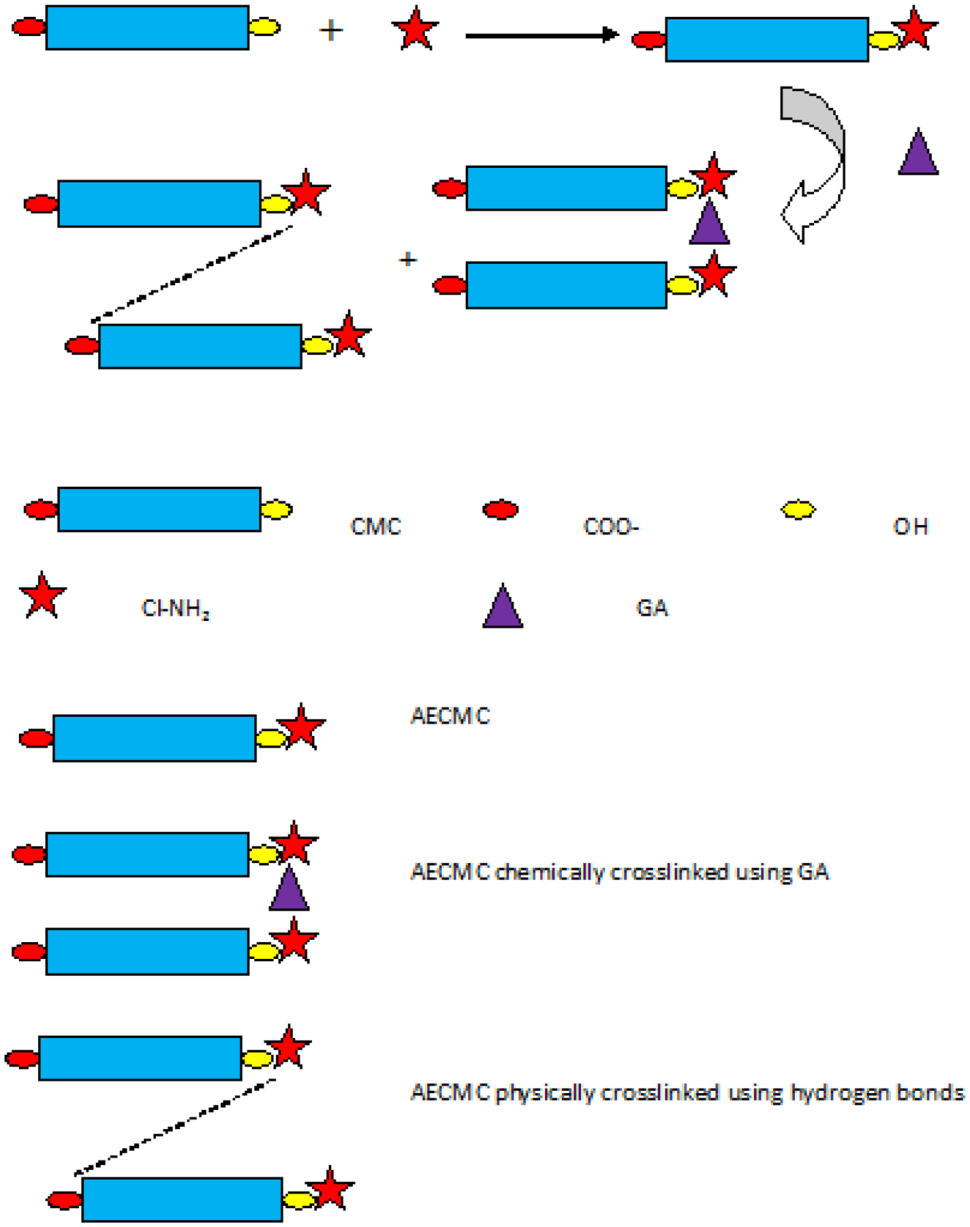
Proposed chemical and physical crosslinking of the AECMC hydrogel adsorbent through the introduced amine groups via Glutaraldehyde and hydrogen bonds.

The obtained relatively low adsorption capacity of the Aminated Ethyl Carboxymethyl Cellulose (AECMC-4) adsorbent could be referred to many reasons summarized as follows:

1. The low number of amine active sites for adsorption,
2. The high number of repulsive adsorption carboxylic groups,
3. It was consumed by the introduced active amine group sites in chemical and physical crosslinking processes with Glutaraldehyde and CMC carboxylic groups as suggested in Fig. 6.

Extensive amination reaction using different alkylated amine chain lengths is needed to introduce more amine groups to active adsorption sites. Further detailed study of the modification process conditions is recommended to optimize the adsorption capacity.

### Kinetic, isotherm, and thermodynamic studies

In this study, three linear kinetic models were employed to characterize the kinetics of the sorption process of MO utilizing AECMC-1, AECMC-2, AECMC-3 adsorbents.

The pseudo-first-order kinetic model^54^:

4 $${\text{ln }}\left( {{\text{q}}_{{\text{e}}} - {\text{q}}_{{\text{t}}} } \right) = {\text{ln q}}_{{\text{e}}} - {\text{k}}_{{1}} {\text{t}}$$

The pseudo second-order rate (chemisorptions)^55^:

5 $${\text{t}}/{\text{q}}_{{\text{t}}} = \left( {{1}/{\text{k}}_{{2}} {\text{q}}_{{\text{e}}}^{{2}} } \right) + {\text{t}}/{\text{q}}_{{\text{e}}}$$

The Elovich model^56^:

6 $${\text{q}}_{{\text{t}}} = \, \alpha \, + \, \beta {\text{ ln t}}$$

**Figure 7:**
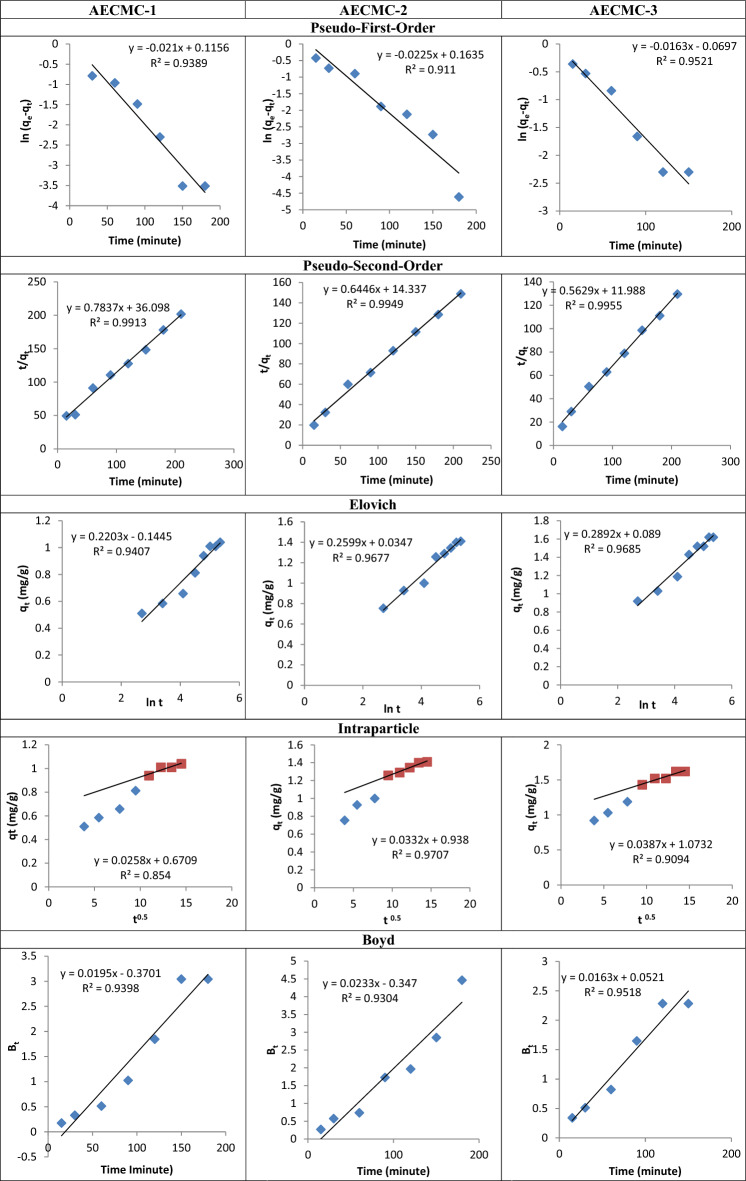
The Pseudo-first order, the Pseudo-second order, Elovich, Intraparticle, and the Boyd kinetic models kinetic model for MO molecules sorption by AECMC-1, AECMC-2, AECMC-3 adsorbents.

The values of the first-order rate constant k1 and correlation coefficient, R^2^, determined from the slope of the plot ln (q_e_ − q_t_) vs time (Fig. 7), are presented in Table 6. The table presented data indicating good correlation coefficients. However, there is a clear difference between the estimated values of q_e_, computed using the equation and the experimental values. This indicates that the model being used is not suitable for accurately describing the sorption process.

**Table 6:** The adsorption parameters of the pseudo-first, the pseudo-second order, Elovich, Intraparticle, and the Boyd kinetic models.

Adsorbent	Pseudo-first-order				Pseudo-second-order			Elovich		
	q_e,exp_ (mg/g)	q_e,cal_ (mg/g)	K_1_ (min^−1^)	R^2^	q_e,cal_ (mg/g)	K_2_ (g/mg^−1^ min^−1^)	R^2^	β (g/mg)	α (mg/g min)	R^2^
AECMC-1	1.04	0.921	− 0.019	0.983	1.277	36.09	0.991	0.22	− 0.144	0.94
AECMC-2	1.41	1.177	− 0.022	0.911	1.553	14.33	0.994	0.259	0.034	0.967
AECMC-3	1.62	0.933	− 0.016	0.952	1.779	11.98	0.995	0.289	0.089	0.968

Adsorbent	Intraparticle diffusion			Boyd						
	k_d_ (mg/g min)	C	R^2^	R^2^						
AECMC-1	0.025	0.67	0.854	0.991						
AECMC-2	0.033	0.938	0.97	0.994						
AECMC-3	0.038	1.073	0.909	0.995						

The experimental result presented in Fig. 7 conforms to the principles of pseudo-second-order kinetics. The values of q_e_, computed, and k2 can be deduced from the slope and intercept of the plot, as depicted in the image. Furthermore, the correlation coefficients, specifically R^2^, were recorded and presented in Table 6. The second-order equation provides a good description of the kinetics of MO molecule sorption onto AECMC-1, AECMC-2, AECMC-3 adsorbents, as evidenced by the high R^2^ value (about 1) obtained from the linear regression analysis of the data presented in the table. Therefore, this implies that the step that controls the rate of sorption in these processes might potentially be chemisorption, which involves the interaction of sorbent and sorbate through the sharing or exchanging of electrons^57^. Furthermore, a comparison was made between the estimated values of q_e_, which were determined from the junction sites of the second-degree reaction kinetic curves (as shown in Table 6), and the q_e_ values acquired from the experimental data. Therefore, the second-order rate expression provides the most satisfactory fit to the data.

The calculated parameters of the Elovich equation from Fig. 7 have been compiled in Table 6. The parameter β represents the number of available sites for removal increase with increasing the ion exchange capacity of the AECMC-1, AECMC-2, AECMC-3 adsorbents in almost a direct linear way, while the parameter α represents the removal quantity when the natural logarithm of time (Ln t) is equal to zero, which corresponds to a time of one hour (equilibrium time). The aforementioned value provides valuable insights into the removal behavior exhibited by the initial step^58^. Nevertheless, the data acquired by the Elovich equation demonstrates a strong correlation with the experimental data, better than the Pseudo-first order model and exhibiting lower fit compared to the Pseudo-second order model.

To address the shortcomings of the aforementioned models in explaining the diffusion mechanism, intraparticle model (Eq. 7) was described the liquid film diffusion or intraparticle diffusion^59^, while the external mass transfer examined by Boyd model (Eq. 8)^60^, are represented as following:

7 $$q_{t} = K_{d} t^{1/2} + C$$

8 $${\rm B}_{t} = - 0.4978 - Ln\left( {1 - q/q_{ \propto } } \right)$$

where q_t_ is the amount of adsorbate at time t, q_e_ is the equilibrium amount of adsorbate, and K is the diffusion rate constant, k_d_ is the intraparticle diffusion rate, and C is the thickness of the boundary layer. The obtained data are presented in Table 6.

Weber and Morris explored the possibility of affecting the adsorption process via Intraparticle diffusion resistance^61^ using the intra-particle diffusion model described in Fig. 7. Two separate linear portions that represent each portion can be observe from the Fig. 7. These two linear portions in the Intraparticle model suggest that the removal process consists of both surface removal and Intraparticle diffusion. While the initial linear part of the plot is the indicator of the boundary layer effect, the second linear portion is due to Intraparticle diffusion^59^. The Intraparticle diffusion rate (k_d_) calculated from the slope of the second linear portion and the values of C, the intercept, provides an idea about the thickness of the boundary layer. The larger the intercept, the higher the boundary layer effect^62^. In the case of involving the Intraparticle diffusion in the sorption process, then a linear relationship would result from the plot of q_t_ versus t^1/2^. Intraparticle diffusion would be the controlling step if this line passed through the origin^63^. Figure 7 confirms straight lines not passed through the origin. The difference between the rate of mass transfer in the initial and final steps of the sorption process may cause the deviation of straight lines from the origin. Accordingly, it can be concluded that pore diffusion is not the sole rate-controlling step^64^. Additional processes, such as the adsorption on the boundary layer, may also be involved in the control of the adsorption rate.

The kinetic expression further analyzed the adsorption data given by Boyd et al.^60^ to characterize the actual rate-controlling step involved in the MO sorption process. The calculated B_t_ values were plotted against time, as shown in Fig. 7. The linearity of this plot will provide useful information to distinguish between external transport and Intraparticle transport controlled rates of sorption. Figure 7 shows the plot of B_t_ versus t, which is a straight line that does not pass through the origin, indicating that film diffusion governs the rate-limiting process^65^.

**Figure 8:**
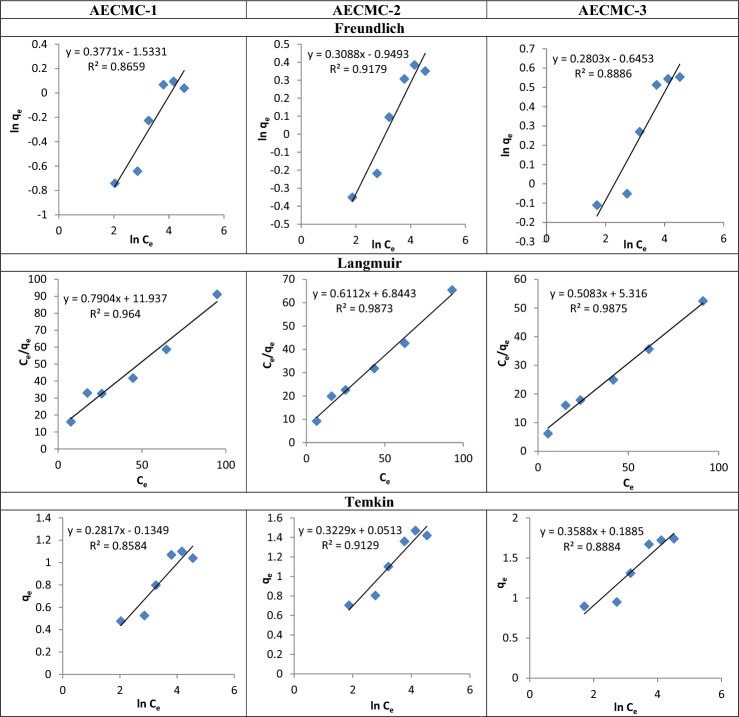
The Freundlich, Langmuir, and the Temkin isotherm models for MO adsorption using AECMC-1, AECMC-2, and AECMC-3 adsorbents.

The Freundlich and Langmuir isotherm models have been employed to characterize the MO adsorption processes^66–68^. Figure 8 presents linear regression analyses of the sorption data about MO compounds using the Freundlich isotherm model. Based on the data presented in the figure, it can be observed that the Freundlich equation accurately predicts a positive correlation between the concentration of MO molecules on the AECMC-1, AECMC-2, and AECMC-3 sorbents and the overall concentration of MO molecules. This alignment with the experimental findings suggests compatibility between the theoretical model and the observed outcomes. Moreover, the Freundlich isotherm was found to be obeyed by the removal of MO molecules, as evidenced by the correlation coefficient (R^2^) values of 0.865, 0.917, and 0.888. The Freundlich constants n_f_ and K_F_ are determined through estimation based on the slope and intercept of the linear plot and tabulated in Table 7. Based on the evaluated value of n_f_, it was determined that n_f_ > 1 indicated a favorable sorption behavior for MO molecules when using the AECMC-1, AECMC-2, and AECMC-3 adsorbents^69^.

**Table 7:** The parameters and the correlation coefficients of the Langmuir, the Freundlich, and the Temkin isotherm models.

Adsorbent	Langmuir isotherm			Freundlich isotherm			Temkin isotherm		
	q_max_ (mg/g)	K_L_ (L mg^−1^)	R^2^	K_f_ (mg/g)	*n*_f_	R^2^	B_T_ (J/mol)	K_T_ (L/g)	R^2^
AECMC-1	1.266	15.1	0.964	0.216	2.653	0.865	0.281	0.621	0.858
AECMC-2	1.637	11.2	0.987	0.387	3.25	0.917	0.322	1.172	0.912
AECMC-3	1.969	10.46	0.987	0.525	3.57	0.888	0.358	1.69	0.888

Figure 8 depicts a linear representation of the Langmuir equation^70^, showcasing the adsorption of MO molecules onto the AECMC-1, AECMC-2, and AECMC-3 adsorbents at different concentrations of starting MO molecules. When plotting the ratio of C_e_ to q_e_ against C_e_, it is expected to observe a linear relationship with a slope equal to 1 divided by qm and an intercept equal to 1 divided by q_m_K. The Langmuir equation demonstrates a strong representation of the sorption process of MO molecules onto the AECMC-1, AECMC-2, and AECMC-3 adsorbents, as seen by the high R^2^ values of 0.964, 0.987, and 0.987. This observation suggests a strong mathematical correlation. The calculated values of q_m_ were determined to be 1.266, 1.637, and 1.969 mg/g, whereas the values of K were discovered to be 15.1, 11.2, and 10.46 L/mg. This suggests that the AECMC-1, AECMC-2, and AECMC-3 adsorbents exhibited an increasing level of efficiency in adsorbing MO molecules and showed a decrement of the sorption energy, indicating a strong affinity between AECMC-1, AECMC-2, and AECMC-3 adsorbents and the MO molecules with increase of the amine groups’ content.

To determine the favorable or unfavorable of the adsorption process, a dimensionless separation factor (R_L_) is employed and computed using Eq. (9)^71^;

9 $${\text{R}}_{{\text{L}}} = {1}/(1 + {\text{KC}}_{0} )$$

The obtained R_L_ values for the adsorption of MO molecules by AECMC-1, AECMC-2, and AECMC-3 adsorbents are ranged from 0.0066 to 0.00066, 0.00888 to 0.00088, and 0.0095 to 0.00095, respectively, indicate favorable adsorption due to their range of 0–1^72,73^. This finding further supports the favorable nature of the Langmuir isotherm about the sorption of MO molecules onto AECMC-1, AECMC-2, and AECMC-3 adsorbents, as observed within the experimental conditions of this work.

The Temkin isotherm^74^ is presented in Fig. 8. The Figure depicts the correlation between the natural logarithm of the equilibrium concentration (ln C_e_) and the quantity of MO molecules adsorbed (q_e_) onto the AECMC-1, AECMC-2, and AECMC-3 adsorbents at different initial concentrations of MO molecules. This graph facilitates the calculation of the isotherm constants B and K_T_, which can be determined from the slope and intercept of the graph, respectively. The determined values of K_T_ are 0.621, 1.172, and 1.69 L/g, which signifies the equilibrium binding constant associated with the highest binding energy. Conversely, constant B, with a value of 0.281, 0.322, and 0.358 J/mol, are linked to the heat of sorption for the AECMC-1, AECMC-2, and AECMC-3 adsorbents, respectively; Table 7.

In conclusion, a comprehensive summary of the R^2^ values acquired from the application of three equilibrium isotherm models to the adsorption of MO molecules on AECMC-1, AECMC-2, and AECMC-3 adsorbents is presented. The Langmuir isotherm model produced the highest coefficient of determination (R^2^ = 0.964, 0.987, and 0.987). This model assumes an entirely homogeneous surface, consisting of a finite number of identical sites. Additionally, it assumes that there is minimal interaction between the adsorbed molecules, leading to the formation of a monolayer throughout the sorption process. In contrast, the Freundlich isotherm exhibited a reasonable coefficient of determination (R^2^ = 0.865, 0.917, and 0.888), indicating a good fit of the model to the adsorption of MO molecules on the polymer. This model takes into account the presence of heterogeneous surface site energies and the formation of several layers of sorption. The Temkin isotherm, which represents a compromise between the Freundlich and Langmuir isotherm models, exhibited R^2^ values of 0.858, 0.912, and 0.888, which is equivalent to that of the Freundlich isotherm.

The values of thermodynamic parameters should be taken into consideration to conclude the spontaneity of the adsorption process. An automatic system will display a decrease in *ΔG*^*o*^ and *ΔH*^*o*^ values with increasing the temperature. All the thermodynamic parameters calculated from the following equations^75,76^.

10 $$ln{K}_{d}=\frac{\Delta S}{R}-\frac{\Delta H}{RT}$$

where: $${K}_{d}=\frac{{q}_{e}}{{C}_{e}}$$

11 $$\Delta G= -RTln{K}_{d}$$

where *R* is the gas constant (8.314 J/mol K), and *T* is the temperature in K.

**Figure 9:**
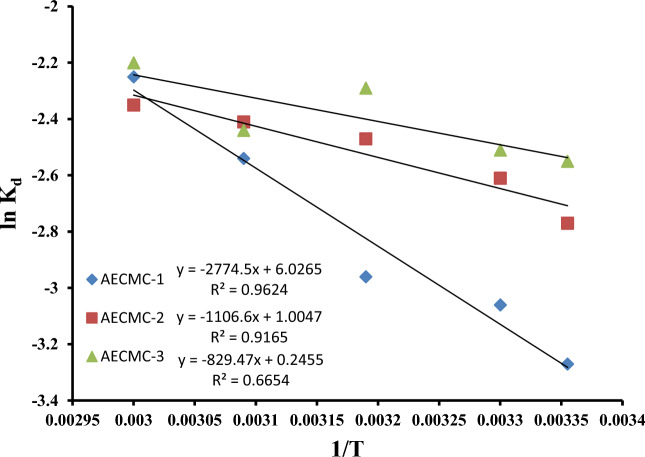
The Van’t Hoff plot of the adsorption of MO using developed AECMC-1, AECMC-2, and AECMC-3 adsorbents.

Table 8 presents the values of the thermodynamic parameters as depicted in Fig. 9. The positive values of ΔH° (23.063, 9.195, and 6.895 kJ/mol) signify that the process is endothermic. This observation can explain the observed rise in the adsorption capacity of MO molecules with increasing temperature. The magnitude of the enthalpy change associated with the chemisorption process, ranging from 40 to 120 kJ mol^−1^, is shown to be more substantial compared to that of the physisorption process. The heat of adsorption values obtained in this study, 23.063, 9.195, and 6.895 kJ mol^−1^, suggest that the adsorption of the MO ions is likely due to physisorption, contradicting the findings of the kinetics study which proposed chemisorption as the mechanism of adsorption. Therefore, it is apparent based on the lower ΔH° value that physisorption is also involved in the adsorption process^75–77^. The MO molecules exhibit adhesion to the adsorbent surface solely through weak intermolecular interactions. The entropy change, ΔS° (50.1, 8.35, and 2.037 J/mol K), demonstrates the increase in disorder at the solid/liquid interface during the adsorption of the MO molecules^75–77^. The values of ΔG° serve as indicators of the thermodynamic feasibility of a given process.

**Table 8:** Thermodynamic parameters values of the MO molecules adsorption on the AECMC-1, AECMC-2, and AECMC-3 adsorbents under different temperatures.

*1/T*	*ΔG* (kJ/mol)	*ΔH* (kJ/mol)	*ΔS* (J/mol^−1^ K^−1^)
AECMC-1			
0.003355	8.101	23.063	50.1
0.0033	7.709		
0.00319	7.703		
0.00309	6.821		
0.003	6.229		
AECMC-2			
0.003355	6.863	9.195	8.35
0.0033	6.575		
0.00319	6.428		
0.00309	6.472		
0.003	6.506		
AECMC-3			
0.003355	6.318	6.895	2.037
0.0033	6.323		
0.00319	5.959		
0.00309	6.525		
0.003	6.091

## Conclusion

Novel amino-ethyl carboxymethyl cellulose crosslinked hydrogel (AECMC) has been developed for the first time by modification of the carboxymethyl cellulose with the 2-chloro-ethyl amine to remove the Methyl orange (MO) azo-dye from dyes contaminated wastewater. The degree of modification was found to increase with increased concentration of 2-chloro-ethyl amine and monitored through evaluation of the induced amine group concentration as ion exchange capacity increased to 7.5 (meq/g). The provided amine groups acted as adsorption-positive charge centers for MO azo-dye anions. The ion exchange capacity directly relates to the MO adsorption percentage and adsorbent capacities. Both parameters were increased with the ion exchange capacity. The kinetic adsorption process was characterized by a high-speed initial stage within the first 15 min, and then the adsorption rate started to slow down until leveling off. The MO concentration positively affects the adsorption capacity, where the highest adsorption capacity (1.86 mg/g) was related to the adsorbent with the highest ion exchange capacity (AECMC-4). It is worth mentioning that part of the immobilized amine groups participated in the crosslinking process. Two crosslinking approaches were expected. The first one is a chemical one through Schiff base formation with Glutaraldehyde, which is a permanent one. The second one is a physical one through the formation of a polyelectrolyte complex between the remaining part of the introduced amine groups and the carboxylic groups of the CMC backbone, which is a temporary one. Competition with the MO anions leads finally to the adsorption process, while the free CMC carboxylic groups repel the MO anions adsorption process. That may explain the low adsorption capacity of the developed AECMC adsorbents. The adsorption temperature positively impacts the adsorption capacity, mainly when a low-aminated CMC derivative is used. That effect is reduced with higher aminated CMC derivatives (AECMC-4). The adsorption capacity of MO is much greater in acidic solutions than in alkaline conditions where a drastic decline was noticed above pH 7.0, especially for the high amine content adsorbents: AECMC-2, AECMC-3, AECMC-4. The adsorption capacity for the developed AECMC adsorbents decreased linearly with increasing the adsorbent dose. The reduction percentage of the adsorption capacities ranged from 12.5% of AECMC-3 to 23% of AECMC-1 adsorbents. Finally, the agitation speed variation from 100 to 250 rpm has almost no significant effect on the adsorption capacity of all the developed AECMC adsorbents. The only exception was found in the case of the AECMC-1 adsorbent, where the adsorption capacity increased by 25% with increased agitation speed within the studied range. Only 16% of the MO removal efficiency was lost after ten cycles of sorption–desorption processes. The AECMC-4 adsorbent has a good sorption–desorption performance and can be used confidently without a noticeable reduction in its sorption capacity to remove MO. The developed AECMC derivatives have been characterized using different characterization techniques such as FTIR, TGA, and DSC. Additionally, the cationic exchange and water uptake capacity have been evaluated and correlated to the adsorption efficiency.

The kinetics of the adsorption process was seen to have a rapid early stage lasting around 15 min, followed by a gradual deceleration in the rate of adsorption until reaching a plateau. The data obtained from the experiment indicated that the process of MO adsorption adhered to the Pseudo-second-order kinetic model. The concentration of MO exhibits a positive correlation with the adsorption capacity, with the adsorbent possessing the highest ion exchange capacity. To understand the adsorption mechanism, various adsorption models were examined, specifically the Intraparticle diffusion models to identify which of these processes is the limiting factor in the rate of adsorption. Additionally, the Boyd model was utilized to determine the precise step that controls the rate of adsorption for the MO ions. The resultant line is straight and does not pass through the origin, showing that the rate of the limiting process is governed by film diffusion. The Langmuir equation demonstrates a strong representation of the sorption process of MO molecules onto the AECMC-1, AECMC-2, and AECMC-3 adsorbents. The values of K were discovered to be 15.1, 11.2, and 10.46 L/mg. This suggests that the AECMC-1, AECMC-2, and AECMC-3 adsorbents exhibited an increasing level of efficiency in adsorbing MO molecules and showed a decrement of the sorption energy, indicating a strong affinity between AECMC-1, AECMC-2, and AECMC-3 adsorbents and the MO molecules with increase of the amine groups’ content. The adsorption capacity is notably enhanced by increasing the adsorption temperature, particularly when employing low aminated carboxymethyl cellulose (CMC) derivative. The aforementioned effect exhibits a decrease in magnitude when utilizing CMC derivatives with higher levels of amination, specifically AECMC-4. The thermodynamic parameters associated with the process of MO adsorption have been investigated at various temperatures of adsorption. The Van't Hoff model was employed for this purpose, revealing that the adsorption process is both spontaneous and endothermic in nature.

## Data Availability

The datasets used and/or analyzed during the current study available from the corresponding author on reasonable request.
